# Relationship Between Vitamin D Serum Levels and the Severity of Atopic Dermatitis—A Mapping Review of Evidence with Emphasis on Geography

**DOI:** 10.3390/jcm15031048

**Published:** 2026-01-28

**Authors:** Marko Vidak, Metka Fišer, Nevena Makaji, Eva Tavčar

**Affiliations:** 1 Institute for Biostatistics and Medical Informatics, Faculty of Medicine, University of Ljubljana, Vrazov trg 2, SI-1000 Ljubljana, Slovenia; 2 Faculty of Pharmacy, University of Ljubljana, Aškerčeva cesta 7, SI-1000 Ljubljana, Sloveniaeva.tavcar@ffa.uni-lj.si (E.T.)

**Keywords:** Vitamin D, atopic dermatitis, latitude, Human Development Index, evidence gap map

## Abstract

Atopic dermatitis (AD) is a chronic inflammatory skin disease with early-age onset. While vitamin D (VitD) has been associated with AD alleviation, geographical factors should be considered as VitD synthesis depends on sunlight exposure and dietary intake. We conducted a mapping review to identify geography-related evidence gaps in interventional and observational studies on the VitD-AD inverse association. We analyzed latitude and the Human Development Index (HDI) as background geographical factors. The review identified 38 studies (17 interventional, 21 observational), of which 26 confirmed the inverse VitD-AD association. Of all reviewed studies, 73% were from latitudes above 35° N, and 70.3% were from developed countries. The median latitude and HDI were 37.5° N and 0.915, respectively. Conversely, only 5.4% of studies were from Africa and 8.1% from Latin America. Studies that did not confirm the inverse VitD-AD association tended to be concentrated in developed countries at higher latitudes (median latitude 42.4° N, median HDI 0.937). Only 8.1% of all studies were from low-latitude developed countries, and among interventional studies this share was even lower (6.3%). In addition, 52.6% of studies lacked data on baseline VitD variability and 13.2% had no baseline VitD data at all. More thorough data reporting and additional clinical studies from countries that do not follow the high latitude/high HDI overlap pattern would facilitate future meta-analyses aimed at clarifying the role of VitD in AD treatment.

## 1. Introduction

Atopic dermatitis (AD) is a chronic inflammatory skin disease with a typical onset in early childhood. Sometimes the disease disappears in adulthood, while adult patients may also go through cycles of remission and relapse [1]. Global AD prevalence rates have been estimated to be 2% for adults and 4% for children, and females had slightly higher prevalence rates than males—2.8% and 2.4%, respectively [2]. The main symptoms of AD are skin rash and itching, usually on the face, neck and extremities, which can affect concentration and sleep. As these parts of the body are usually visible, the disease is also a social burden [3]. The severity of AD symptoms is determined by two indices: Scoring Atopic Dermatitis (SCORAD) and Eczema Area and Severity Index (EASI). The main difference between these two indices is that EASI only includes clinical factors in the index calculation, while SCORAD also includes the AD patient’s self-assessment to determine the psychological burden of the disease on the patient’s quality of life [4].

Vitamin D (VitD) is an essential nutrient that plays a critical role in bone health by maintaining calcium and phosphate homeostasis. Other physiological functions of vitamin D include controlling cell proliferation and differentiation, regulating thyroid hormone secretion, stimulating insulin synthesis, modulating the immune system and regulating blood pressure (5). The biologically active form of VitD (1,25-dihydroxy-vitamin D) is synthesized in the human organism in several steps from precursor substances that are ingested with food. The final step of the synthesis takes place in the skin and requires exposure to ultraviolet β-radiation (UVB) [5,6]. The serum concentration of the biologically active form is difficult to measure. Therefore, one of its precursors, 25-hydroxyvitamin D (25(OH)D), is used as a surrogate for the analytical determination of serum VitD concentration (7), and VitD deficiency occurs when the serum concentration of 25(OH)D is below 20 ng/mL [7,8].

Based on published reviews, VitD may be beneficial for patients with several immune diseases, including AD [9,10,11]. However, beneficial effects cannot occur if serum concentrations of VitD are too low. Due to the mechanism of VitD synthesis in the human body, limited exposure to sunlight may contribute to VitD deficiency. Low sunlight or UVB exposure, typical of winters at high latitudes (above 35° from the Equator in both directions), is generally insufficient for normal VitD synthesis. Above latitude 35°, winter UVB irradiance does not reach the minimum radiation level (20 mJ/cm^2^) required to generate previtamin D3 from its precursor [12]. Thus, even in sunny climates at latitudes above 35°, winter sunlight exposure does not facilitate endogenous VitD synthesis. Reduced endogenous VitD synthesis can also occur in climatic conditions where sunlight exposure is limited regardless of latitude or season, such as during persistent cloudiness or extreme cold or heat, which both force people to stay indoors. Lack of UVB exposure may lead to VitD deficiency if not compensated by dietary intake or supplementation [13].

This is where factors of social geography, such as standard of living, should be considered. Options for dietary intake and supplementation of VitD during winter months are generally better in economies with a higher standard of living. This standard can be quantitatively measured using composite statistical indices, such as the Human Development Index (HDI). The HDI is calculated from variables related to per capita income, life expectancy, and educational attainment. Life expectancy and education are included to balance the importance of economic indicators. The index values range from 0 to 1, with higher values indicating a higher level of human development. Based on HDI scores, countries are categorized into four levels of development: very high, high, medium, and low, with the threshold for a very high level of development set at 0.800. This level includes the entire Western world but is not identical to it, as there is also a growing number of non-Western countries with a very high HDI, particularly in East Asia [14].

The scope of this mapping review was to identify geography-related knowledge gaps, specifically combinations of background geographical factors that are underrepresented in existing studies and where further research on VitD and AD in these settings is needed. We identified original interventional and observational studies that analyzed the association between VitD serum concentration and AD severity and determined whether these studies confirmed the inverse association between VitD and AD. We also determined the geographical background of the reviewed studies, including the latitudes of study locations and the HDI values of the countries where the studies were conducted.

## 2. Methods

### 2.1. Literature Search

Our methodology followed the Preferred Reporting Items for Systematic Reviews and Meta-Analyses—Scoping Review Extension (PRISMA-ScR) checklist (Supplementary Table S1), as scoping reviews are closely related to mapping reviews [15]. The review protocol was registered at the Faculty of Pharmacy, University of Ljubljana, in 2023. Four reviewers with medical or pharmaceutical backgrounds independently conducted the search between February and August 2025, with the final query performed on August 25, 2025.

We conducted our search in the databases MEDLINE (using the PubMed search engine [16]), EMBASE, and Web of Science. As the PRISMA-ScR protocol recommends a detailed description of the search for one of the included databases, we provide it here for MEDLINE. The reviewers first identified the terms used in the controlled vocabulary of Medical Subject Headings (MeSH) [17] for VitD (Vitamin D) and AD (Dermatitis, Atopic). These MeSH terms were then incorporated in the following query: “Dermatitis, Atopic” [Mesh] AND “Vitamin D” [Mesh] AND (“1995/01/01” [Date—Publication]: “2024/12/31” [Date—Publication]). Equivalent queries were built for other analyzed databases.

The identified bibliographical entries from all databases were compiled, and duplicates were removed, leaving only unique entries. Recently published review articles (published on or after 1 January 2018) among these entries were included for browsing reference lists using the snowballing technique. This approach was used because some relevant original research articles might appear in the reference lists but may not be identified by database query algorithms.

All unique bibliographical entries, including those discovered through snowballing, were then assessed for eligibility by retrieving and reading their abstracts. Entries were considered eligible if they met the following condition:

- The article investigates the direct association between a person’s VitD serum levels and the severity of their AD symptoms.

The articles that met this condition were further screened for eligibility through full-text review. The following inclusion criteria were applied:

- The study was conducted on humans.
- The article was written in English.
- The article reported the results of a completed study.

Articles meeting these criteria were included in the mapping review. Relevant data from the included studies were recorded in Microsoft Excel spreadsheets (Microsoft Corporation, Redmond, WA, USA) as research data. The reviewers independently determined whether the studies confirmed the inverse association between VitD serum levels and AD severity, using information from the conclusions or abstracts of the reviewed studies. Disagreements between reviewers were resolved through consultation until consensus was reached.

### 2.2. Identification of Evidence Gaps

The following geographical factors were used for the evidence-mapping analysis:

- Latitude: Whether the absolute latitude (either north or south of the Equator) is higher than 35° (high latitude) or lower than/equal to 35° (low latitude).
- HDI: Very high HDI compared to the other three levels of human development (high, medium, and low). The 2023 HDI values were used, as they were the latest available data at the time of writing in 2025, and HDI values of 0.800 or higher were considered very high. Hong Kong’s separate HDI values were considered, as the UN lists this entity apart from mainland China. The HDI data were taken from the UNDP Human Development Database [18]. Within the group with very high HDI we identified the subgroup of core Western countries using the overlap between the UNDP category of the most developed countries and the academic definition of the Western world [19]. A country or region was considered part of core West if it was included according to both criteria. The core West included Northern and Western Europe, the United States, Canada, Australia, and New Zealand.

In addition, study type was considered (interventional vs. observational) as it might affect study outcomes. Interventional studies included clinically supervised VitD supplementation, while observational studies examined only the relationship between VitD serum concentration and AD severity. Missing information in the reported data on study outcomes was analyzed, as data gaps limit the potential for data reuse in future systematic reviews or meta-analyses.

Microsoft Excel’s pivot table function was used for the evidence map gap presentation, and Excel’s graph functions were used for the diagrams. The Drawio application was used for the literature review flowchart, and the maps were created with the MapChart application.

## 3. Results and Discussion

### 3.1. Identification of Clinical Studies and Data Extraction

**Figure 1:**
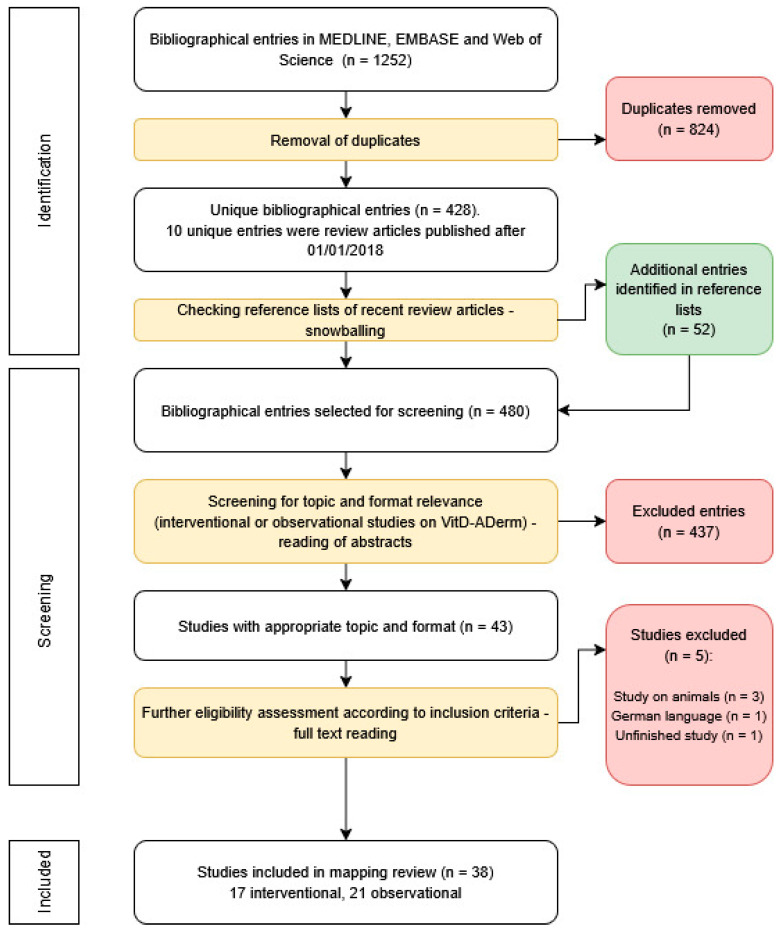
PRISMA flowchart of literature search. *n* = number of studies.

The literature search workflow is shown in Figure 1. The initial database search, along with browsing the reference lists of recent review articles, yielded 480 unique bibliographical entries after duplicates were removed. These entries were screened for topic and format eligibility, resulting in 43 appropriate articles. These were further screened for additional inclusion criteria, and ultimately 38 original research articles on VitD-AD studies were included in the mapping review: 17 interventional studies with VitD supplementation and 21 observational studies.

The clinical data from the reviewed studies are listed in Table 1.

This mapping review of VitD and AD included a relatively large number of interventional and observational studies, whereas previous reviews (most of which were meta-analyses) tended to apply strict exclusion criteria that reduced sample size. A typical meta-analysis on this topic includes only interventional studies that report the net change in AD severity indices after VitD supplementation, specifically the difference in SCORAD or EASI change between the test and control groups. In contrast, a mapping review is not limited to studies that report such numerical data, as study outcomes are not assessed statistically but through qualitative text analysis. To our knowledge, it is also the first study to examine the influence of social geographical factors as reflected in HDI values. In our mapping review, 26 of 38 studies (68.4%) confirmed the efficacy of VitD. These findings are consistent with previous meta-analyses, in which a supermajority of included studies confirmed the efficacy of VitD in relieving AD [9,11,58]. However, these meta-analyses have mostly focused on interventional studies, even though their sample sizes are usually smaller than those of observational studies. In our review, the observational studies included a cumulative total of 4800 AD patients (an average of 240 per study), while the intervention studies included 568 patients in their test groups (an average of 33.4 per study). Although observational studies provide larger sample sizes, the smaller, controlled environment of interventional studies is crucial for establishing causality.

Some possible intervening factors were not sufficiently controlled in all the reviewed interventional studies, e.g., sun exposure. In addition, two interventional studies (11.8% of all interventional studies) did not mention whether established corticosteroid therapy was still allowed during VitD supplementation. Other key information was also missing, e.g., two (11.8%) interventional studies and three (14.3%) observational studies did not include information on the mean or median age of the included patients. Most of the reviewed studies were conducted on children as only nine studies (23.7%) reported the average or median age higher than 18 years.

As interventional studies are more focused on AD severity outcomes (usually measured with SCORAD or EASI), it is not surprising that only 10 of 17 interventional studies (58.8%) included data on VitD concentrations before and after the intervention with VitD supplementation (Table 1). Samochocki et al. [32] included post-intervention data for a subgroup of the original sample that included only the most severely deficient patients. This subgroup had a mean VitD concentration of 7.43 ng/mL before the intervention, which increased to 13.05 ng/mL after the intervention. The intervention increased the mean VitD serum concentration in all 10 studies with complete data. In addition, post-intervention concentrations exceeded the threshold for deficiency (20 ng/mL), in all 10 cases with complete data, but remained below the 30 ng/mL level in 3 studies (30%). 10 interventional studies (58.8% of all interventional studies) reported no VitD data for the control groups, which leads to risk of bias due to selective reporting.

Baseline data on mean serum VitD concentrations were reported by 33 of 38 studies (86.8%). One interventional study that reported severe baseline VitD deficiency in Mongolia [23] was not included in this count because no data on average VitD levels were provided, only the proportion of study participants with deficient levels. Only 18 studies (47.4%) in total (6 interventional—35.3%, and 12 observational—57.1%) reported standard deviations in baseline VitD levels (Table 1).

Most reviewed interventional studies (14/17—82.4%) used an experimental design with a single dosing regimen of VitD supplementation for all test subjects, regardless of the severity of their AD, baseline VitD levels, or age. Cholecalciferol was used as the form of VitD for supplementation, except in one study, which used ergocalciferol [34]. Daily doses of VitD ranged from 1000 to 10,000 international units (IU), and intervention durations ranged from 3 weeks to 3 months, with the largest number of studies (6/17—35.3%) using a 3-month intervention. Durations were reported in different time units (days, weeks, and months), which are difficult to convert into one another. The IU has been defined by the World Health Organization (WHO) based on in vivo activity in model animals to enable comparison of the biological activity of different substances. One IU of cholecalciferol is equivalent to 25 ng or 65 pmol [59]. Most of the reviewed interventional studies (11 studies—64.7%) used daily doses in the 1000–2000 IU range.

One interventional study [35] used different dosing regimens based on AD severity groups, defined by baseline SCORAD values. While SCORAD values of 25 and 50 are conventionally used to define AD severity groups (mild AD: SCORAD < 25; moderate AD: 25 ≤ SCORAD ≤ 50; severe AD: SCORAD > 50), this study used 40 as the cut-off point between severe and mild AD, and patients with severe AD received twice the daily dose of VitD. Another interventional study used age 12 as the cut-off value, and test subjects above that age received double the daily dose of VitD [20]. A three-month interventional study from Curitiba, Brazil, administered an additional 5000 IU per day to patients with baseline VitD deficiency (serum level < 20 ng/mL) during the first four weeks of the intervention [28]. While this interventional study reported a significant improvement in AD severity following vitamin D intervention, a preceding observational study from the same city and research team did not find a significant correlation between baseline VitD levels and AD severity [54]. Among the reviewed observational studies, we also noted diverging definitions of AD severity classes. Two observational studies used the SCORAD value of 40 as the border between severe and moderate AD while maintaining 25 as the moderate/mild border [43,55]. Another observational study only compared VitD levels between AD patients and non-patients, without dividing patients based on AD severity [48].

VitD levels were mostly reported in ng/mL, and only one observational study used nmol/L [39], although nmol/L is part of the International System of Units (SI), while ng/mL is not. Atopic dermatitis (AD) severity was mostly expressed using SCORAD composite index values, which were used by 11 of 17 interventional studies (64.7%) and 12 of 21 observational studies (57.1%). EASI was used in four interventional studies, while objective SCORAD was used in one interventional and two observational studies. One interventional study used cathelicidin expression as the main biomarker of AD severity [27]. Objective SCORAD is a modification of the original composite index that excludes the two subjective items (pruritus and sleeplessness). Objective SCORAD values are not comparable with the full SCORAD unless data on the grades for each individual SCORAD item are available [60]. SCORAD is also not directly comparable with EASI, although one study reported a high positive correlation between the two composite indices (Spearman correlation coefficient of 0.92). However, the two indices diverged in the assessment of some AD symptoms, such as xerosis and oozing [61]. In addition to SCORAD and EASI, some reviewed studies used complementary composite indicators of AD severity, such as the Patient-Oriented Eczema Measure (POEM) and Investigator’s Global Assessment (IGA) indices [20,23].

The lack of data on average or median values of VitD and AD severity indices, as well as their corresponding variabilities, limits the possibilities of statistical analysis with fixed or random effect models and therefore restricts comparative interpretation. Consequently, the absence of these statistical parameters renders a significant portion of existing research unsuitable for meta-analytical pooling, effectively wasting valuable clinical data and preventing precise effect size estimation.

### 3.2. Geographical Background of the Reviewed Studies

Table 2 presents the geographical background of the reviewed studies and their outcomes regarding the inverse association between VitD and AD.

**Figure 2:**
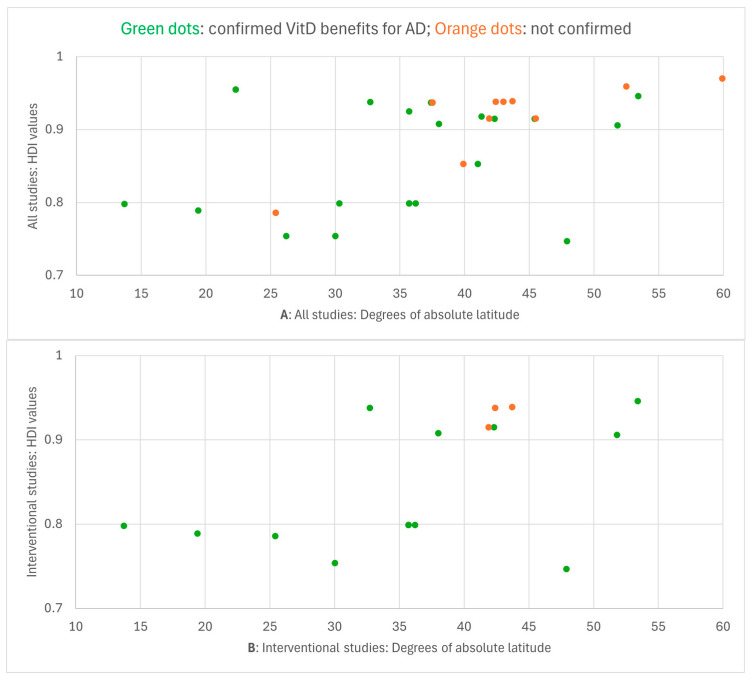

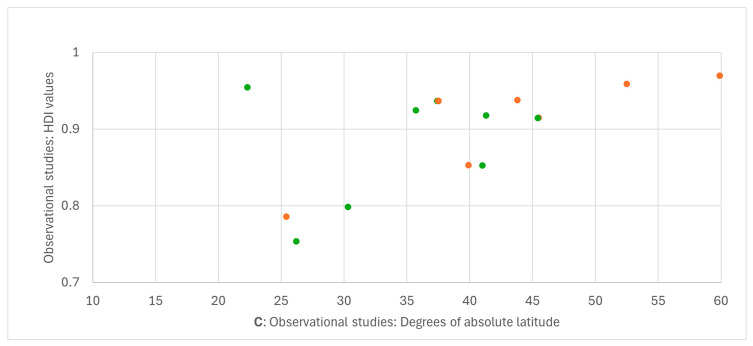
Distribution of reviewed studies by absolute latitude (degrees north or south of the Equator) of locations where studies were conducted, and by HDI values of countries in which studies were conducted. Green dots indicate studies that confirmed the inverse VitD-AD association, while orange dots indicate studies that did not confirm this association. (**A**): with all studies included, (**B**): with only interventional studies included, (**C**): with only observational studies included.

Figure 2 presents the data from Table 3, mapping all studies by both latitude and HDI. One study (Hata et al. 2014 [26]) was excluded because it was a multicenter study conducted at locations with latitudes ranging from 32.7° N to 45.5° N, thus covering both high-latitude and low-latitude settings.

Among all the studies included, 27 (73.0%) were from latitudes above 35° N, and 26 (70.3%) were from developed countries with very high HDI values (0.800 or higher). The median latitude and HDI for all 37 included studies were 37.5° N and 0.915, respectively. Interventional studies are more relevant for AD treatment, as they involve clinically supervised VitD supplementation administered to AD patients. Considering only the 16 interventional studies, the median latitude is 37.1° N and the median HDI is 0.8525. Eleven interventional studies (68.8%) were conducted at latitudes above 35° N, and eight (50.0%) were from countries with very high HDI values. These numbers and proportions for interventional studies are somewhat better than those for all studies, indicating a less skewed global distribution of interventional studies. Observational studies, which provide weaker and more circumstantial evidence for VitD benefits in AD treatment, were also largely conducted at high latitudes (≥35° N): 16 out of 21 included observational studies (76.2%) were from these latitudes, with a median latitude of 37.5° N. Additionally, 18 of 21 observational studies (85.7%) were conducted in countries with very high HDI, and the median HDI was 0.937. These data for observational studies confirm the overrepresentation of high-latitude developed countries in VitD-AD research. This geographic clustering suggests that current clinical consensus is heavily derived from specific environmental conditions (low UVB in winter, very high human development), potentially limiting the relevance of these findings to populations living in different climates and economies.

Several included studies were conducted in cities near the 35° latitude threshold on either side. However, all studies performed at latitudes just above 35° were conducted in cities with relatively cold winter climates for their latitude, such as Tehran and Sabzevar in northern Iran, Tokyo, and the Seoul area. Thus, their winter climatic conditions resemble those at higher latitudes. In contrast, cities just below the 35° threshold all have sunny climates typical of subtropical latitudes, such as San Diego, Cairo, and Kerman in southern Iran.

**Figure 3:**
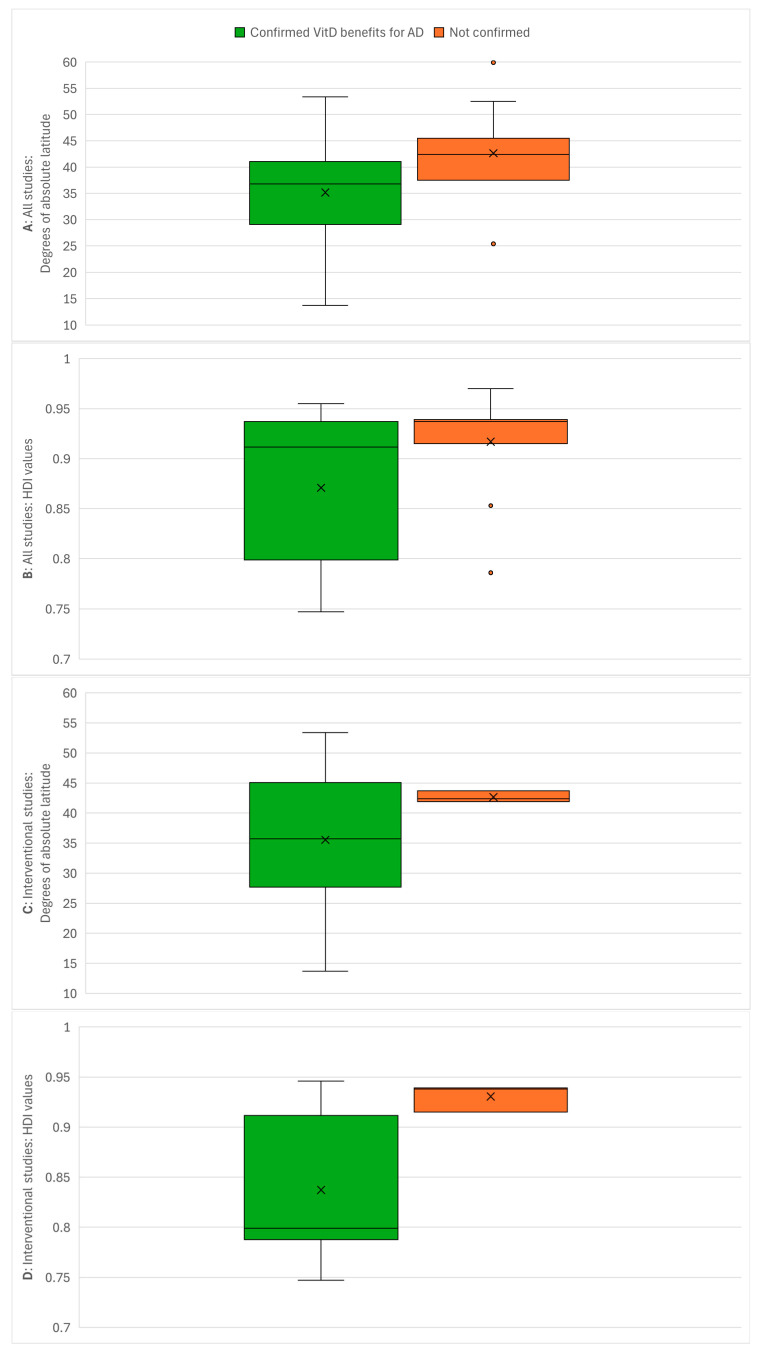

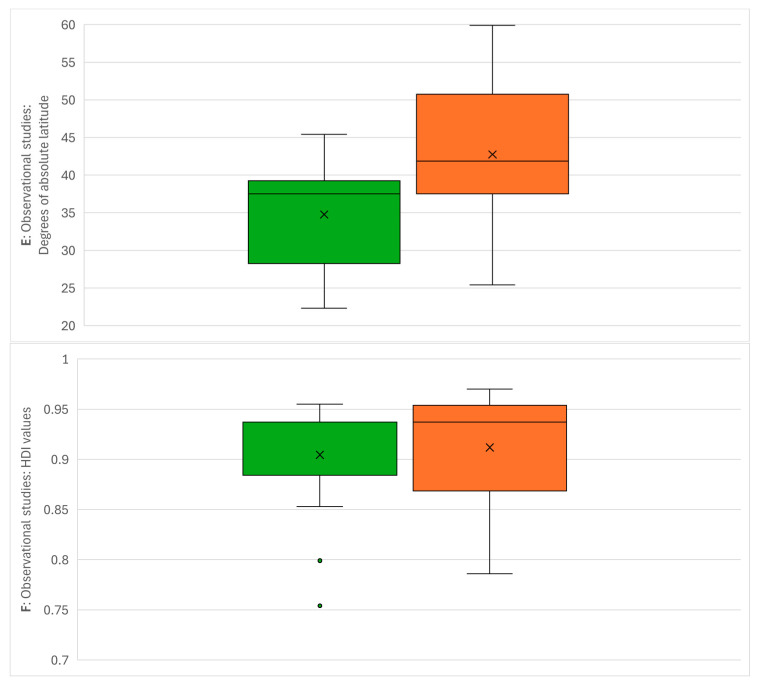
Comparison of latitude/HDI values between the studies that confirmed the inverse VitD-AD association (green) and the studies that did not confirm it (orange). (**A**): Comparison of absolute latitudes (degrees north or south of the Equator) of locations where studies were conducted with all studies included, (**B**): comparison of HDI values of countries in which studies were conducted with all studies included, (**C**): comparison of absolute latitudes of locations where studies were conducted with only interventional studies included, (**D**): comparison of HDI values of countries in which studies were conducted with only interventional studies included, (**E**): comparison of absolute latitudes of locations where studies were conducted with only observational studies included, (**F**): comparison of HDI values of countries in which studies were conducted with only observational studies included. Labels: x, arithmetic mean; ◦, outlier.

Figure 3 separately presents the distribution of latitudes and HDI values for studies that confirmed the inverse VitD-AD association and for studies that did not confirm it. The data are shown for all studies, interventional studies, and observational studies.

Eleven included studies did not confirm the inverse VitD-AD association, and these studies were predominantly clustered in developed countries at higher latitudes. Ten (90.9%) were from latitudes above 35° N, and the same number and percentage (10; 90.9%) were from developed countries. The median latitude and HDI for non-confirming studies were 42.4° N and 0.937, respectively, compared to 37.5° N and 0.915 for all 37 included studies. The latitude range was 59.9° N to 25.4° S (with the latter representing a geographical outlier from Brazil), while the HDI range was 0.970 to 0.786 (with the latter also an outlier from Brazil). Only three non-confirming interventional studies were identified, with a latitude range of 43.7° N to 41.9° N and an HDI range of 0.939 to 0.915. The eight non-confirming observational studies had a median latitude of 41.85° N and a median HDI of 0.937, compared to 37.5° N and 0.937 for all observational studies. The ranges for non-confirming observational studies were 59.9° N to 25.4° S for latitude and 0.970 to 0.786 for HDI. The distinct concentration of non-confirming results in high-HDI, high-latitude regions suggests that environmental or lifestyle factors specific to these areas—such as widespread food fortification or supplementation habits—may be masking the therapeutic effects of VitD intervention.

### 3.3. Geography-Focused Evidence Gap Map

Table 3 presents the distribution of reviewed studies to map evidence gaps. One study (Hata et al. 2014 [26]) was excluded because it was conducted at multiple locations both above and below the 35° latitude threshold, making it impossible to classify within a single bracket. The data are presented separately for all studies (*n* = 37), interventional studies (*n* = 16), and observational studies (*n* = 21).

**Table 3:** Distribution of reviewed studies by geographical background and study outcomes.

All Studies (*n* = 37)				
Geographical Background		Number of Studies	Inverse Association VitD-AD	
			Confirmed	Not Confirmed
1	High latitude, HDI ≥ 0.8, core West	11	4	7
2	High latitude, HDI ≥ 0.8, other developed	12	9	3
3	High latitude, HDI < 0.8	4	4	0
4	Low latitude, HDI ≥ 0.8, core West	1	1	0
5	Low latitude, HDI ≥ 0.8, other developed	2	2	0
6	Low latitude, HDI < 0.8	7	6	1
Interventional studies (*n* = 16)				
1	High latitude, HDI ≥ 0.8, core West	5	2	3
2	High latitude, HDI ≥ 0.8, other developed	2	2	0
3	High latitude, HDI < 0.8	4	4	0
4	Low latitude, HDI ≥ 0.8, core West	1	1	0
5	Low latitude, HDI ≥ 0.8, other developed	0	0	0
6	Low latitude, HDI < 0.8	4	4	0
Observational studies (*n* = 21)				
1	High latitude, HDI ≥ 0.8, core West	6	2	4
2	High latitude, HDI ≥ 0.8, other developed	10	7	3
3	High latitude, HDI < 0.8	0	0	0
4	Low latitude, HDI ≥ 0.8, core West	0	0	0
5	Low latitude, HDI ≥ 0.8, other developed	2	2	0
6	Low latitude, HDI < 0.8	3	2	1

Abbreviations: AD, atopic dermatitis; VitD, vitamin D.

**Figure 4:**
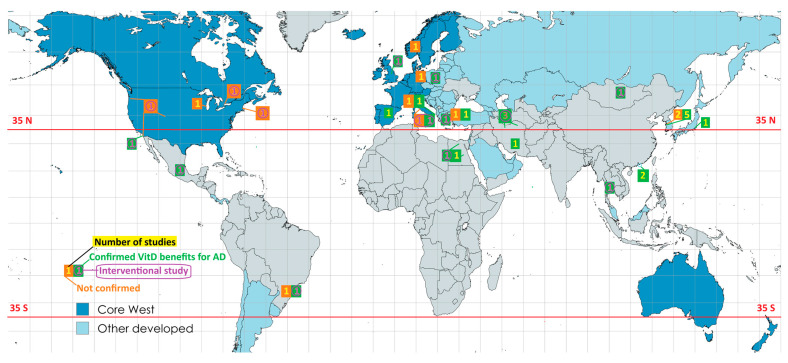
Geographical distribution of all reviewed studies. Green signs indicate studies that confirmed the inverse VitD-AD association, while orange signs indicate studies that did not confirm it. Numbers in violet indicate interventional studies.

Among the 37 included studies, 12 (32.4%) were conducted in Western countries. In contrast, only 2 (5.4%) studies were from Africa and 3 (8.1%) from Latin America. Western countries were overrepresented among the 11 studies that did not confirm the VitD-AD inverse relationship, accounting for 7 studies (63.6%). Due to the overlap between high latitude and high HDI, more data from countries that do not fit this pattern would be needed for future meta-analyses. However, only 3 (8.1%) studies were from low-latitude developed countries, and among interventional studies, this proportion was even lower (1 of 16 studies; 6.3%). High-latitude developing countries represent the opposite case and were slightly more represented (4 of 37 studies; 10.8%). All studies from high-latitude developing countries were interventional. Of the 16 interventional studies, 6 (37.5%) were from Western countries, and all three interventional studies that did not confirm the inverse VitD-AD association were from the West. Among the 21 observational studies included, 6 (28.6%) were from Western countries, but among the 8 observational studies that did not confirm the inverse VitD-AD association, the Western share was 50.0% (4 of 8 studies). Notably, the Western high-latitude category was the only one in which non-confirming studies outnumbered those that confirmed VitD benefits for AD. In this category, the ratios favored non-confirming studies regardless of study type (3:2 for interventional, 4:2 for observational, and 7:4 for all studies). The data from Table 3 are visualized on the maps in Figure 4.

Geographical analysis is rarely included in review articles. To the best of our knowledge, we found only one review article that analyzed geographical factors in clinical studies—the meta-analysis by Ng and Yew [10], which compared test groups with healthy controls based on selected factors such as latitude (above or below 35°) and continent. Another review highlighted AD as a disease with ethnic-dependent heterogeneity of immunophenotypes, while existing therapeutic approaches with biological drugs are mainly tailored to only one of these phenotypes (T-helper type 2 dominant). As the reviewed interventional and observational studies from Western countries rarely reported the ethnic background or skin phototype of included test subjects, their results may not be representative of minority populations [62,63]. A study on children of South Asian origin in the United Kingdom (outside the scope of this review) demonstrated a high frequency of winter VitD deficiency and severe AD in this ethnic minority [64]. Including diverse populations in research could therefore facilitate the development of targeted AD treatments. One of the reviewed interventional studies from the United States was conducted on a sample that was majority (73%) non-white [34]. However, this study did not report significant alleviation of AD after one month of VitD supplementation. Cultural differences in clothing practices represent another possible confounding factor, as they affect sun exposure. For example, wearing a full-face veil in some Muslim cultures may reduce sun exposure, but this clothing practice does not apply to children, who are the main group of AD patients.

As the reviewed interventional studies generally allowed uncontrolled exposure to sunlight during VitD supplementation, one would expect that abundant sunlight at low latitudes would obscure possible beneficial effects of supplemental VitD, as sunlight exposure enables endogenous VitD synthesis (even for control group members who did not receive supplementation). However, all three interventional studies that did not confirm VitD benefits for AD treatment were from high-latitude Western countries (latitude range 41.9–43.7° N). One possible explanation could be the observed higher baseline serum concentrations of VitD in Western patients. The literature indicates that the benefits of VitD for AD patients are weaker for those who already have serum VitD levels above the deficiency range, i.e., above 20 ng/mL [58,65]. The median baseline VitD serum concentration of 10 Western studies (out of 12 total—excluding Hata et al. 2014 [26], which was not included in the evidence gap mapping, and Sidbury et al. 2008 [34], which did not report VitD data) was 23.84 ng/mL. Only one of these 10 studies reported a mean baseline VitD concentration slightly below 20 ng/mL (19.4 ng/mL). In Europe, the average vitamin D serum concentration actually increases with latitude rather than decreases as expected [7]. This phenomenon can be attributed to food fortification and supplementation measures, which are common in Northern European countries. In addition, public awareness campaigns promote compliance with supplementation programs [66]. Supplementation interventions particularly target children, as the disease often occurs already in early childhood [67].

This review provides a methodological analysis of data from interventional studies, including baseline and final data on VitD levels and AD severity, as well as daily dose and duration of VitD intervention. For future VitD–AD clinical studies, researchers may use this review to improve data reporting—particularly by reporting baseline and final averages or medians and their corresponding variabilities—streamline study designs to minimize confounding factors (such as avoiding different dosing regimens based on AD severity groups, avoiding concomitant corticosteroid use, and controlling for sun exposure if solar UVB radiation during the intervention exceeds the threshold for endogenous VitD synthesis), and select intervention protocols that are comparable to the majority of previous studies in terms of intervention duration and daily VitD dose.

## 4. Conclusions

The analysis of background geographical factors revealed an overlap between high latitude and a high level of human development. As both latitude and standard of living may affect baseline VitD levels, it is difficult to determine which has a greater influence on VitD and, consequently, on the usefulness of its supplementation for AD. The evidence gap mapping therefore highlighted the need to conduct VitD–AD studies—particularly interventional studies with clinically supervised and controlled VitD supplementation—in locations that do not follow the latitude–HDI pattern. This includes both developed countries at low latitudes, such as Singapore and most of Australia, as well as developing countries at high latitudes, such as some Central Asian countries. In addition, we noted a lack of reported data on VitD serum levels and AD severity, such as means or medians and their corresponding standard deviations or interquartile ranges. In particular, the absence of variability data poses an obstacle for future systematic reviews and meta-analyses, such as analyses of global variations in baseline VitD or baseline AD severity among AD patients. The use of mutually incomparable indices for AD severity, such as SCORAD and EASI, further limits the possibility of reusing data for statistical comparison. In conclusion, we emphasize the importance of data management to ensure the findability, accessibility, interoperability, and reusability of research data, as this facilitates the reuse of research data for reviews and meta-analyses. The key missing data in some of the reviewed interventional studies are VitD levels in the control groups, as well as the variability of reported VitD levels and AD severity indices. Addressing these geographical and methodological blind spots would enable future authoritative meta-analyses that would clarify the role of VitD in AD treatment.

## Figures and Tables

**Table 1:** Summary of the reviewed studies—clinical data.

Article	Average Age [Years]	Sample Size	Average Baseline Serum VitD [ng/mL]	Variability of Baseline Serum VitD [ng/mL]	Average Baseline AD Severity	Daily VitD Dose [IU] and Duration [Months] of VitD Supplementation	Average Final Serum VitD [ng/mL]	Average Final AD Severity
Interventional studies								
Albenali LH 2016 [20]	11	12	24.4 (median)	25 (IR)	No data	6000 or 10,000 IU/1 month	No data	Mean SCORAD reduction of 42% *
Aldaghi M 2020 [21]	0.37	27	No data	No data	34.4	1000/2	No data	Share of patients with severe SCORAD (>50) decreased (7.4%–> 3.7%) *
Amestejani M 2012 [22]	23.34	29	9.1	1.3	24.8	1600/2	22.15	15.3 *
Camargo CA Jr 2014 [23]	9 (median)	57	No data	No data	21 (EASI)	1000/1	No data	14.5 (EASI) *
Di Filippo P 2015 [24]	4	22	22.97	8.03	46.13	1000/3	29.41	22.57 *
Galli E 2015 [25]	6.8 (median)	41	56	53.5	12.2	2000/3	105.9	12 (no significant SCORAD reduction)
Hata TR 2014 [26]	31.2	15	28.4	No data	No data	6000/0.67	37.8	No significant EASI reduction
Hata TR 2008 [27]	No data	14	22.5 (median)	No data	No data	4000/0.67	35.5 (median)	Significant reduction in cathelicidin expression *
Imoto RR 2021 [28]	6.6 (median)	152	23.7	No data	19.4	2143/2.9	35.9	12.3 *
Javanbakht MH 2011 [29]	21.2	23	No data	No data	No data	1600/2	No data	Mean SCORAD reduction of 34.8% *
Lara-Corrales I 2019 [30]	7.4	21	25.04	11.12	27.3	2000/3	32.6	15.4 (no significant SCORAD reduction)
Mansour NO 2020 [31]	12 (median)	44	22.8	No data	44.4 (EASI)	1600/3	36.11	20.4 (EASI) *
Samochocki Z 2013 [32]	29.9	20	23.05	12.83	37.1 (objective SCORAD)	2000/3	13.05 (partial result)	20.9 (objective SCORAD) *
Sanchez-Armendariz K 2018 [33]	12.6	29	21.3	6.7	41.3	5000/3	58.5	20.1 *
Sidbury R 2008 [34]	7 (median)	5	No data	No data	10-18.6 (EASI range)	1000/1	No data	No significant EASI change
Tsotra K 2023 [35]	No data	47	No data	No data	49.7 and 62.1	1200 or 2400/2	No data	3.9 and 5.3 *
Udompataikul M 2015 [36]	8.28	10	17.03	13.70–20.20 (range)	18.23	2000/0.9	25.06	8.05 *
Observational studies								
Akan A 2013 [37]	2.75 (median)	73	11.2 (median)	9.2 (IR)	36.8	NA		
Baek JH 2014 [38]	0.66	168	18.3 (median)	24.2 (IR)	27.9 **			
Berents TL 2016 [39]	24.56	449	23.28	No data	No data			
Cheng HM 2014 [40]	35.5	392	16.54	6.93	No data **			
Cheon BR 2015 [41]	6 (median)	91	23.1	2.34	27.7 **			
Chiu YE 2013 [42]	5	94	24.84	6–58 (range)	31.48 (objective SCORAD)			
D’Auria E 2017 [43]	6.2	52	19.4	7.3	33.6			
El Taieb MA 2013 [44]	6.1	29	5.4	1.9	21.5 **			
Farajzadeh S 2015 [45]	5.49	57	24.62	6.71	No data **			
Gilaberte Y 2015 [46]	5.7	114	29.7	15.0	38.7 (objective SCORAD) **			
Han TY 2015 [47]	9.5	72	12.43	4.66	No data **			
Heimbeck I 2013 [48]	No data	1363	20.24	No data	No data			
Kanda N 2012 [49]	38.3	26	4.51 (median)	No data	27.6 **			
Kang JW 2016 [50]	44.9	No data	16.2	0.8	No data **			
Leung TF 2013 [51]	No data	499	8.70	No data	No data **			
Noh S 2014 [52]	20.8	82	10.3 (median)	No data	No data			
Peroni DG 2011 [53]	5.6	37	29.61	10.72	13.4 **			
Robl R 2016 [54]	6.3 (median)	105	21.7 (median)	No data	27.9			
Su O 2017 [55]	8.37	60	16.13	6.72	No data **			
Wang SS 2014 [56]	10.5	498	11.56	6.12	No data **			
Yang AR 2016 [57]	No data	539	17.76	5.84	No data			

Notes: Articles are listed by first author. Sample size for interventional studies includes only test groups with AD patients. Baseline and final values are averages or medians reported in the studies. Variability of baseline VitD levels is measured with standard deviation, and AD severity is measured with the SCORAD index unless stated otherwise in brackets. An asterisk (*) denotes an interventional study that confirmed the benefits of VitD supplementation for AD alleviation. A double asterisk (**) denotes an observational study that confirmed the inverse relationship between VitD serum levels and AD severity. Abbreviations: IR, interquartile range; IU, international units.

**Table 2:** Summary of the reviewed studies—geographical background and study outcomes.

Article	City	Country or Region	Latitude	Country’s HDI (2023)	Group Based on Geographical Background	Confirmed Inverse Association VitD-AD
Berents TL 2016 [39]	Oslo	Norway	59.9 N	0.970	1	No
Albenali LH 2016 [20] *	Sheffield	United Kingdom	53.4 N	0.946	1	Yes
Heimbeck I 2013 [48]	Berlin	Germany	52.5 N	0.959	1	No
Samochocki Z 2013 [32] *	Lodz	Poland	51.8 N	0.906	2	Yes
Camargo CA Jr 2014 [23] *	Ulaanbaatar	Mongolia	47.9 N	0.747	3	Yes
D’Auria E 2017 [43]	Milan	Italy	45.5 N	0.915	1	No
Peroni DG 2011 [53]	Verona	Italy	45.4 N	0.915	1	Yes
Lara-Corrales I 2019 [30] *	Toronto	Canada	43.7 N	0.939	1	No
Chiu YE 2013 [42]	Milwaukee	United States	43.0 N	0.938	1	No
Sidbury R 2008 [34] *	Boston	United States	42.4 N	0.938	1	No
Di Filippo P 2015 [24] *	Chieti	Italy	42.3 N	0.915	1	Yes
Galli E 2015 [25] *	Rome	Italy	41.9 N	0.915	1	No
Gilaberte Y 2015 [46]	Madrid and Huesca	Spain	40.4 N; 42.1 N	0.918	1	Yes
Su O 2017 [55]	Istanbul	Turkey	41.0 N	0.853	2	Yes
Akan A 2013 [37]	Ankara	Turkey	39.9 N	0.853	2	No
Hata TR 2014 [26] *	San Diego, Denver and Portland (Oregon)	United States	32.7 N; 39.7 N; 45.5 N	0.938	1 (Denver and Portland), 4 (San Diego)	No
Tsotra K 2023 [35] *	Athens	Greece	38.0 N	0.908	2	Yes
Cheng HM 2014 [40]	Seoul	South Korea	37.5 N	0.937	2	Yes
Cheon BR 2015 [41]	Seoul	South Korea	37.5 N	0.937	2	Yes
Han TY 2015 [47]	Seoul	South Korea	37.5 N	0.937	2	Yes
Kang JW 2016 [50]	Seoul	South Korea	37.5 N	0.937	2	Yes
Noh S 2014 [52]	Seoul	South Korea	37.5 N	0.937	2	No
Yang AR 2016 [57]	Seoul	South Korea	37.5 N	0.937	2	No
Baek JH 2014 [38]	Seongnam	South Korea	37.4 N	0.937	2	Yes
Aldaghi M 2020 [21] *	Sabzevar	Iran	36.2 N	0.799	3	Yes
Amestejani M 2012 [22] *	Tehran	Iran	35.7 N	0.799	3	Yes
Javanbakht MH 2011 [29] *	Tehran	Iran	35.7 N	0.799	3	Yes
Kanda N 2012 [49]	Tokyo	Japan	35.7 N	0.925	2	Yes
Hata TR 2008 [27] *	San Diego	United States	32.7 N	0.938	4	Yes
Farajzadeh S 2015 [45]	Kerman	Iran	30.3 N	0.799	6	Yes
Mansour NO 2020 [31] *	Cairo	Egypt	30.0 N	0.754	6	Yes
El Taieb MA 2013 [44]	Qena	Egypt	26.2 N	0.754	6	Yes
Leung TF 2013 [51]	Hong Kong	Hong Kong SAR	22.3 N	0.955	5	Yes
Wang SS 2014 [56]	Hong Kong	Hong Kong SAR	22.3 N	0.955	5	Yes
Sánchez-Armendáriz K 2018 [33] *	Mexico City	Mexico	19.4 N	0.789	6	Yes
Udompataikul M 2015 [36] *	Bangkok	Thailand	13.7 N	0.798	6	Yes
Imoto RR 2021 [28] *	Curitiba	Brazil	25.4 S	0.786	6	Yes
Robl R 2016 [54]	Curitiba	Brazil	25.4 S	0.786	6	No

Notes: Groups based on geographical background: 1—High latitude (35 or more degrees from the Equator), HDI ≥ 0.8, core West; 2—High latitude, HDI ≥ 0.8, other developed; 3—High latitude, HDI < 0.8; 4—Low latitude (less than 35 degrees from the Equator), HDI ≥ 0.8, core West; 5—Low latitude, HDI ≥ 0.8, other developed; 6—Low latitude, HDI < 0.8. An asterisk (*) denotes an interventional study. Abbreviations: HDI, Human Development Index; SAR—Special Autonomous Region (of China).

## Data Availability

The datasets generated during and/or analyzed during the current study are available in the Digital Commons Data repository, Vitamin D serum concentration, atopic dermatitis and geographical factors—review—Mendeley Data (accessed on 27 December 2025).

## Supplementary Materials

The following supporting information can be downloaded at: https://www.mdpi.com/article/10.3390/jcm15031048/s1, Table S1: PRISMA-ScR checklist.
